# PSYCHOLOGICAL WELL-BEING IN OLDER ADULTS AFTER MULTIPLE SEVERE WEATHER EVENTS

**DOI:** 10.1093/geroni/igac059.2651

**Published:** 2022-12-20

**Authors:** Katie Cherry, Matthew Calamia, Emily Elliott, Luke Miller, Laura Sampson, Sandro Galea

**Affiliations:** Louisiana State University, Baton Rouge, Louisiana, United States; Louisiana State University, Baton Rouge, Louisiana, United States; Louisiana State University, Baton Rouge, Louisiana, United States; Louisiana State University, Baton Rouge, Louisiana, United States; Harvard T.H. Chan School of Public Health, Boston, Massachusetts, United States; Boston University, Boston, Massachusetts, United States

## Abstract

Catastrophic hurricanes and flooding threaten health and well-being, although the long-term consequences of these events for survivors are poorly understood. In 2005, Hurricane Katrina devastated the US Gulf Coast. Many lost homes in these storms and relocated permanently inland. In August of 2016, historic flooding in Baton Rouge, Louisiana devastated a 22-parish (county) region, resulting in widespread destruction and a second round of disaster-related losses for those who relocated to Baton Rouge after Katrina. The present research is part of a larger longitudinal study on health and well-being after multiple disasters. Cherry et al. (2021) reported that greater flood damage was associated with more symptoms of depression and post-traumatic stress during the Wave 1 immediate impact phase. Here we examined symptoms of depression, anxiety and post-traumatic stress at Wave 2, a follow-up assessment that occurred 9 (+/- 3) months after Wave 1 testing. Three flood exposure groups were compared: non-flooded (controls), single disaster (flooded in 2016) and double disaster (flooded in 2005 and again in 2016). Results indicated that symptoms of depression and post-traumatic stress, which were elevated at Wave 1 for the single and double disaster groups relative to the non-flooded controls, were reduced at Wave 2 and did not differ from the controls. Correlation analyses revealed that age was negatively associated with symptoms of post-traumatic stress, depression, and anxiety, consistent with the inoculation view of post disaster psychological reactions. Implications of these data for understanding older adults’ psychological health after multiple disaster exposures are discussed.

